# Absence of Falciform Ligament Found During Laparoscopic Surgery: A Case Report

**DOI:** 10.7759/cureus.57459

**Published:** 2024-04-02

**Authors:** Nicole A Gonzalez, Michael Rejzer, Rupa Seetharamaiah

**Affiliations:** 1 Surgery, Florida International University, Herbert Wertheim College of Medicine, Miami, USA

**Keywords:** congenital variantion, conventional laparoscopic cholecystectomy, round ligament, hepatic ligament, falciform ligament

## Abstract

Anatomic variants of hepatic ligaments are rare, and complications attributable to these variants may be difficult to diagnose. Our aim is to contribute to the literature surrounding the incidental finding of a congenital absence of the falciform ligament. We report the case of a 37-year-old man who underwent a laparoscopic cholecystectomy for acute cholecystitis. During the operation, the patient was noted to have an apparent absence of the falciform ligament attachment to the liver. The round ligament was attached from the liver to the anterior abdominal wall at the level of the umbilicus. The round ligament is inserted into the inferior surface of the liver as a thick, cordlike structure encased in fat. In rare cases, the small intestine can pass through a falciform ligament defect and become trapped while remaining within the peritoneal cavity, leading to difficult-to-diagnose internal hernias. This condition can lead to intestinal obstruction, incarceration, and strangulation. This directed our decision to divide the remaining round ligament at the liver and close to the abdominal wall. When defects of hepatic ligaments are found incidentally during laparoscopic surgery, these investigators recommend that the operating surgeon consider dividing the remaining ligament as a protective procedure to prevent complications such as internal hernias, intestinal obstruction, incarceration, and strangulation.

## Introduction

Anatomic variants of the hepatic ligaments are rare, and most are clinically insignificant [1,2]. In an observational analysis of 1,802 consecutive patients who underwent laparoscopic surgery from 1981 to 1984, partial defects of the falciform ligament were observed in 0.3% of cases [2]. However, complications involving iatrogenic or congenital defects of the hepatic ligaments, such as internal hernias, are difficult to diagnose. These complications can be associated with significant morbidity and mortality if the small intestine becomes obstructed, incarcerated, or strangulated within the hernia [3-6]. There are no current standards of care that guide the surgical management of this condition. Our aim is to contribute to the literature surrounding the incidental finding of a congenital absence of the falciform ligament and report on an intraoperative intervention that was completed to prevent future pathology. This case report was written as per the Surgical Case Report (SCARE) 2020 Guidelines [7].

## Case presentation

A 37-year-old Hispanic male presented to the emergency department with a one-day history of sudden-onset abdominal pain and nonbilious, nonbloody vomiting following a fatty meal. The pain, rated at seven out of 10 in severity, was described as constant and localized to the right upper quadrant without radiation. The patient had a past medical history of hypertension and a past surgical history of an appendectomy. There was no personal or family history of congenital conditions. He was not taking any medications.

In the emergency department, the patient was found to be afebrile with mild leukocytosis and normal liver enzymes. Abdominal and pelvic computed tomography (CT) with intravenous contrast demonstrated homogeneous enhancement of the liver as well as a distended and thickened gallbladder with cholelithiasis. A follow-up hepatobiliary paraisopropyl-iminodiacetic acid (PIPIDA) scan showed nonvisualization of the gallbladder, supporting a diagnosis of acute cholecystitis. Given the patient’s clinical presentation and workup results, a laparoscopic cholecystectomy was performed.

**Figure 1:**
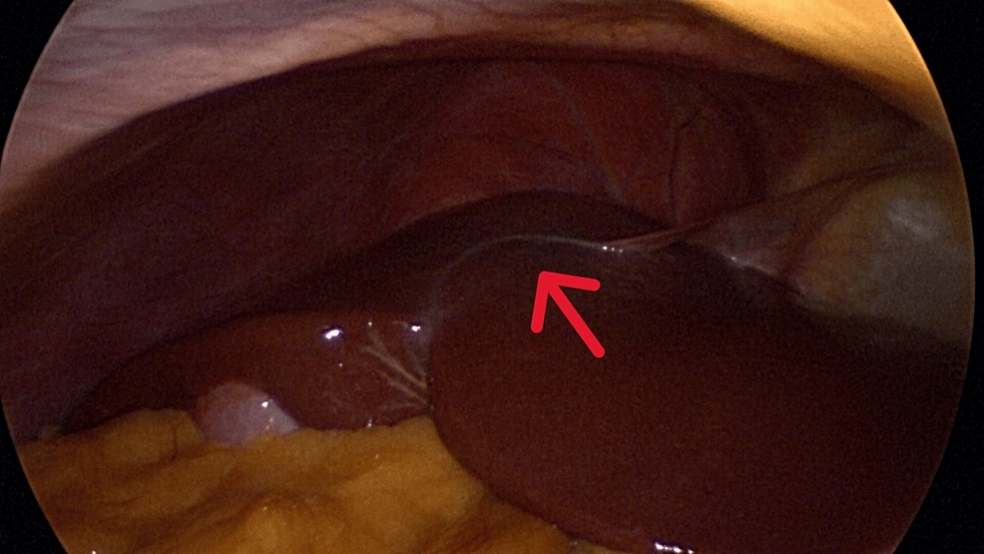
Absence of normal falciform attachment between the right and left lobes of the liver

**Figure 2:**
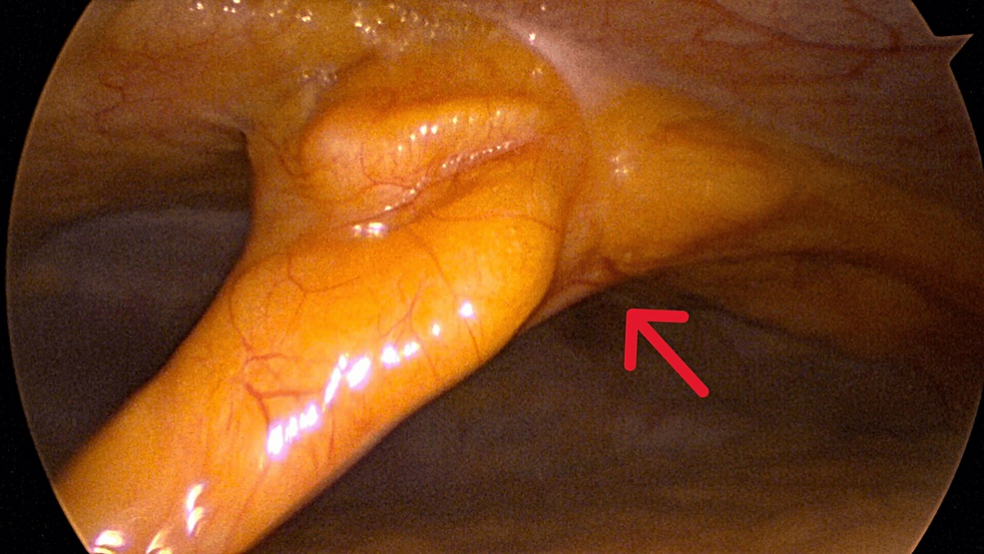
Insertion of the round ligament at the umbilicus on the ventral wall of the abdomen

**Figure 3:**
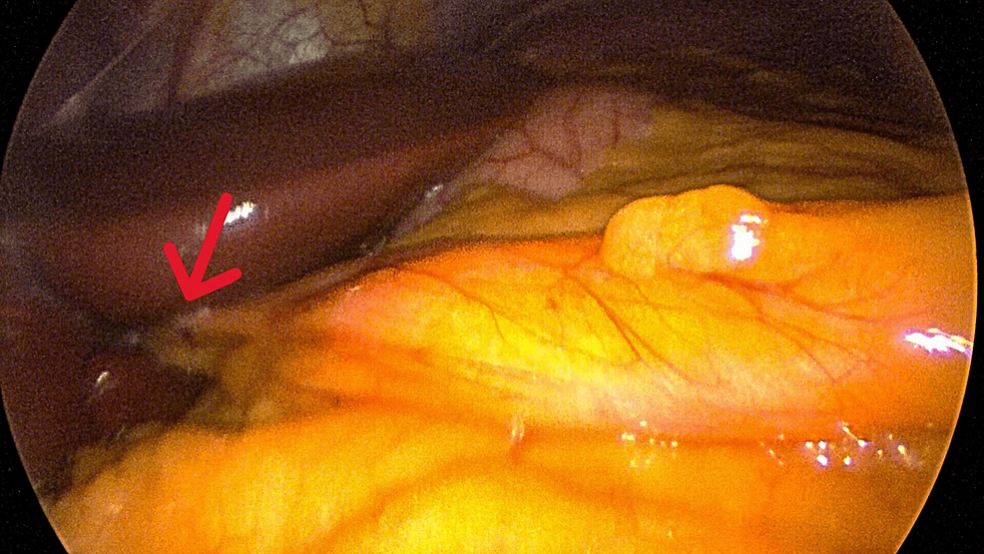
Insertion of the round ligament to the inferior surface of the liver

**Figure 4:**
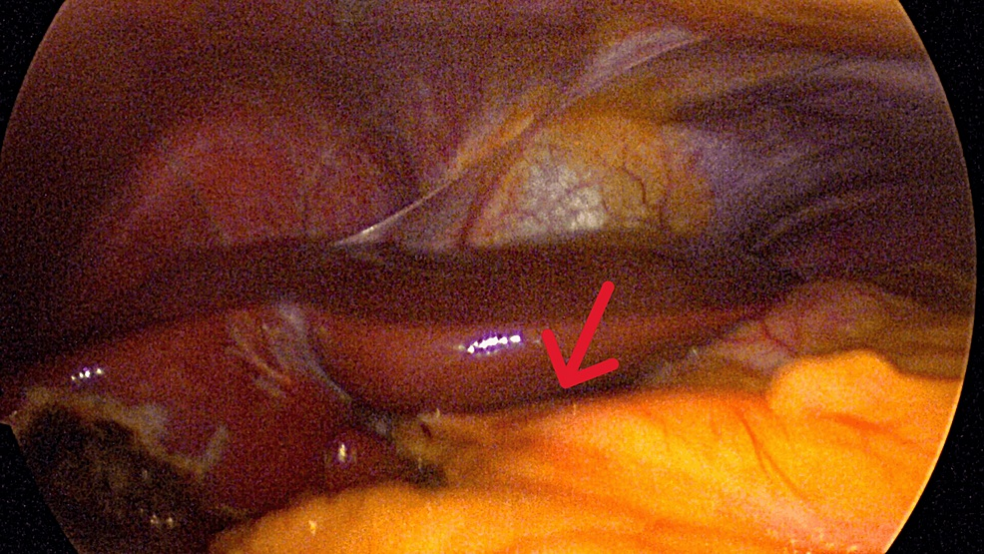
Cordlike round ligament covered in fat

An initial supraumbilical 12 mm balloon trocar was placed in the supraumbilical area using the Hassan technique. This allowed for direct visualization of the peritoneal cavity and viscera. The patient was noted to have an apparent absence of the falciform ligament attachment to the liver (Figure 1). The round ligament of the liver was attached to the anterior abdominal wall at the level of the umbilicus with insertion into the inferior surface of the liver as a thick, cordlike structure encased in fat (Figures 2-4).

 

Additionally, the gallbladder was seen to be intrahepatic with readily identifiable inflammation and distention. Laparoscopic cholecystectomy was completed without any complications.

**Figure 5:**
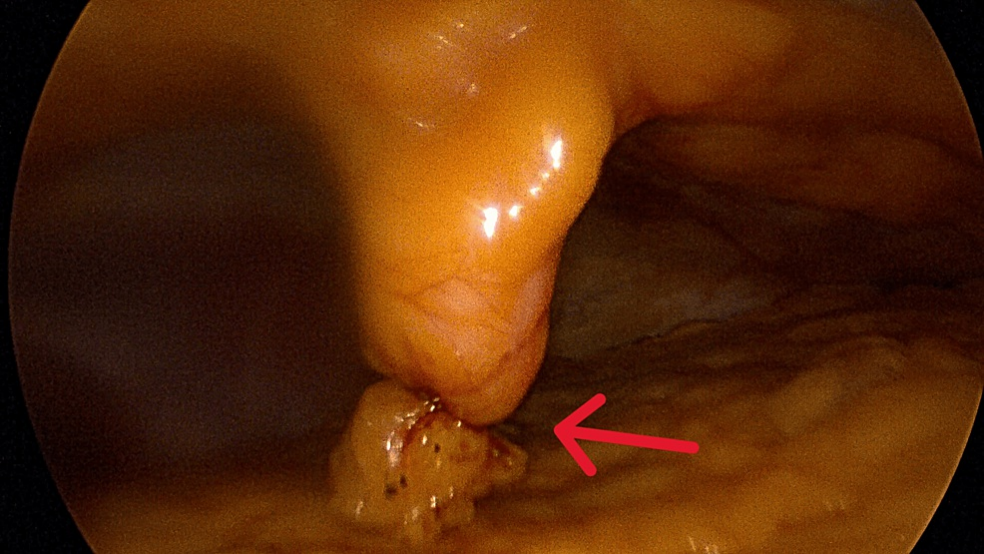
Remaining round ligament divided at the umbilicus

**Figure 6:**
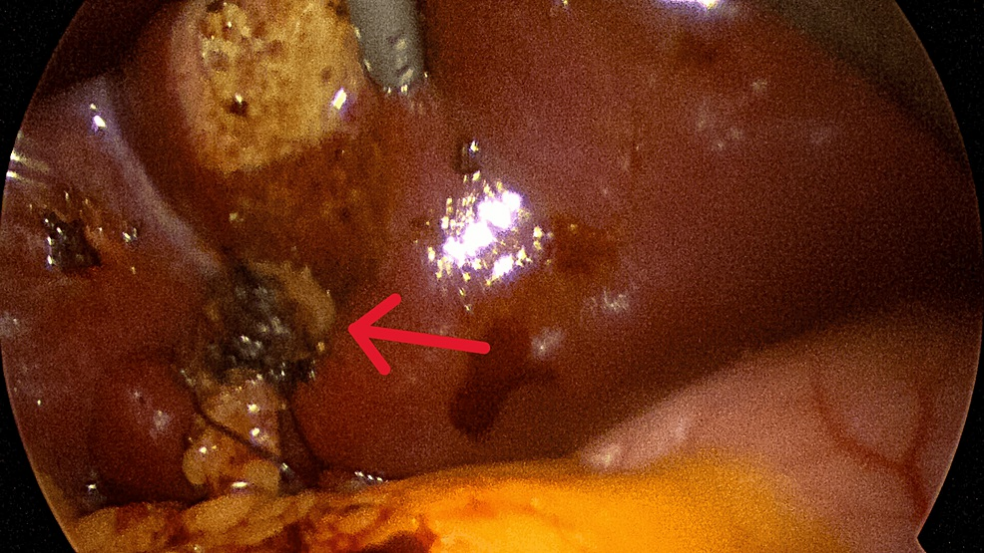
Remaining round ligament divided at the liver

At this point, it was decided to divide the round ligament at both the liver surface and as close to the abdominal wall as possible to prevent potential future complications with internal hernias (Figures 5, 6). The proximal and distal ends were then ligated with a Vicryl Endoloop, and the divided ligament was removed as a specimen. Adequate hemostasis was obtained. Appropriate trocar site closure was performed.

The postoperative period was uneventful. The patient was discharged home on postoperative day one. Postoperative management instructions were explained to the patient. The patient was advised to avoid heavy lifting for three to four weeks and avoid submerging in water for two weeks. At the postoperative visit, the patient was noted to be doing well without any complaints. The pathology of the operative specimens was unsurprising, with the resected gallbladder demonstrating cholelithiasis and signs of acute cholecystitis and the divided round ligament consisting of fibroadipose tissue and mesothelium.

## Discussion

The falciform ligament is a double-layered extension of the parietal peritoneum derived from the embryological foregut’s ventral mesentery. It connects the liver to the ventral abdominal walls and divides the liver into the right and left lobes. The free margin of the falciform ligament contains the round ligament of the liver, or the ligamentum teres hepatis [1].

Physiological variants of the falciform ligament are rare. In an observational analysis of 1,802 consecutive patients who underwent laparoscopic surgery from 1981 to 1984, partial defects of the falciform ligament were observed in 0.3% of cases [2]. Most often, these variants are due to congenital anomalies, but cases of iatrogenic defects in the falciform ligament secondary to surgical procedures have been reported [8,9]. As in this report, these variations may be discovered incidentally during an unrelated procedure. If other organs within the peritoneal cavity do not become involved with the defect, patients may never develop complications of these variations. However, in rare cases, complications do occur. The small intestine can pass through a falciform defect and become trapped while remaining within the peritoneal cavity, leading to intestinal obstruction, incarceration, and strangulation [3-6]. It is estimated that 2% of intestinal obstructions are caused by internal hernias [10], and falciform ligament hernias account for 0.2% of internal hernias [11]. Although extremely uncommon, falciform ligament defects should be made aware as a cause of intestinal obstruction.

The rarity of hepatic ligament defects in conjunction with the vague clinical presentation of a patient with an internal hernia can make this etiology a diagnostic challenge. On clinical examination, these patients commonly present with acute abdominal pain [3,5,6]. Abdominal CT scan can be helpful in confirming the presence of an obstruction [4-6]. However, a literature review of 37 patients with falciform hernia found that only five cases of falciform hernia were correctly diagnosed by preoperative imaging. About 43% of patients required bowel resection, and mortality was 12% [8]. In two case reports of falciform hernias, preoperative imaging showed small bowel obstruction; the culprit of obstruction was then found through diagnostic laparoscopy [5,6].

This case is unique in that current literature provides minimal direction on what intervention to provide, if any, when incidentally finding a hepatic ligament defect during laparoscopic surgery. Keeping in mind the existing literature of complications stemming from these defects, we have provided a protective intervention by dividing the remaining ligament during surgery to prevent the development of internal herniation. Verbal consent was provided by the patient for the publication of this case report.

## Conclusions

In conclusion, this case report sheds light on an unusual anatomical variation of the falciform ligament found intraoperatively, which was an incidental finding unrelated to the patient’s clinical presentation. However, variations of the falciform ligament may cause internal hernias, leading to difficult-to-diagnose small bowel obstructions and compromise of the small intestine. The risk of developing this condition directed our decision to divide the remaining round ligament at the liver and close to the abdominal wall. When defects of the hepatic ligaments are found incidentally during laparoscopic surgery, we recommend that the operating surgeon consider dividing the remaining ligament as a protective procedure to prevent complications such as internal hernias.
